# Bridging jurisdictions and legislatures: an LLM ensemble and arbiter framework for automated legal risk triage in digital media

**DOI:** 10.3389/frai.2026.1841104

**Published:** 2026-07-02

**Authors:** Khrystyna Lipianina-Honcharenko, Tetiana Drakokhrust, Pavlo Bykovyy, Kyrylo Turchynov, Ihor Ihnatiev

**Affiliations:** 1 Department for Information Computer Systems and Control, West Ukrainian National University, Ternopil, Ukraine; 2 Department of Theory of Law and Constitutionalism, West Ukrainian National University, Ternopil, Ukraine; 3 Department of Cyber Security, West Ukrainian National University, Ternopil, Ukraine

**Keywords:** auditability, cross-jurisdiction compliance, human-in-the-loop, legal risk triage, LLM ensemble, risk governance, digital media

## Abstract

**Introduction:**

Autonomous and semi-automated AI tools are increasingly involved in digital-media workflows, creating a need for reliable preliminary screening of legal risks before publication or moderation decisions are made.

**Methods:**

This study presents an LLM Ensemble and Arbiter framework for cross-jurisdiction legal risk triage in digital media. The framework combines: (i) jurisdiction-specific legal grounding through a structured legal knowledge base (LawKB; 61 legal cards covering Ukrainian and EU norms), (ii) five heterogeneous open-weight LLMs, and (iii) a reproducible arbitration policy based on median risk aggregation and disagreement monitoring as a trigger for human review. The pipeline generates separate UA and EU risk assessments, a conservative global verdict, and a traceable rationales to support auditability.

**Results:**

The framework was evaluated on 288 news items with lawyer validation across 576 jurisdiction-specific records. In the global mode, the system achieved a mean absolute error of 0.86 and a confidence-weighted mean absolute error of 0.63, with an approval rate of 95.8 and 95.1% accuracy in selecting the best-performing model rationale, while limiting missed CRITICAL-level cases to 6.3%.

**Discussion:**

These results indicate that the proposed workflow can support conservative, inspectable, and human-supervised legal risk triage across two jurisdictions. Rather than replacing legal judgment, the framework functions as a preliminary decision-support mechanism that strengthens transparency, structured escalation, auditability, and procedural accountability in high-risk digital-media environments.

## 1 Introduction

The rapid integration of artificial intelligence (AI) into digital journalism is reshaping the architecture of legal responsibility in the media sphere. Editorial decisions are increasingly supported or partially replaced by automated content generation, recommendation algorithms, and real-time moderation systems. This shift signals a broader transformation in the paradigm of legal scholarship itself: from retrospective, manual analysis of case law to predictive, algorithmically supported risk screening capable of operating at the speed of digital publication. In this new environment, legal compliance is no longer only a matter of ex post judicial review and increasingly becomes an ex ante challenge of technological design integrated into content production pipelines.

This transformation generates a structural problem of “legal information overload.” Digital media actors must simultaneously take into account national constitutional standards, sector-specific media regulation, platform governance rules, soft-law recommendations, and rapidly evolving case law. For Ukrainian media operating under martial law, the regulatory environment is further complicated by additional national security restrictions, particularly concerning the dissemination of sensitive military or strategic information. At the same time, transnational digital distribution subjects these actors to European regulatory frameworks, most notably the Digital Services Act (DSA), which establishes due diligence obligations, transparency requirements, and systemic risk mitigation duties for online intermediaries and platforms.

The coexistence of Ukrainian emergency-related restrictions, implemented within the framework of national legislation under the supervision of the Verkhovna Rada of Ukraine, and EU-level standards of digital governance adopted by the European Parliament, creates a complex normative intersection. Parliamentary oversight in both jurisdictions plays a decisive role in defining the boundaries of lawful expression, democratic accountability, and the proportionality of restrictions. In Ukraine, parliamentary supervision ensures that wartime information measures remain grounded in constitutional principles and subject to legislative scrutiny. Within the EU, parliamentary involvement in the adoption and monitoring of the DSA underscores the importance of procedural transparency, the protection of fundamental rights, and oversight over executive authorities and digital platforms.

However, despite the growing interdependence between media technologies and public-law regulation, existing research reveals a significant gap. Current studies typically address either (1) AI-based content moderation from a technological perspective, (2) the liability of digital platforms within the framework of EU law, or (3) restrictions on access to information related to national security under states of emergency. The development of an integrated operational framework capable of translating multi-jurisdictional legal standards—particularly those emerging from parliamentary regulation and oversight—into machine-readable, verifiable, and operationally usable compliance mechanisms remains insufficiently explored. In other words, normative principles exist at the legislative level, yet structured methodologies for embedding these principles into automated decision-support systems used by media organizations are lacking.

This article proposes the Ensemble & Arbiter Architecture—a reproducible pipeline for automated legal risk triage in digital media. The framework combines three procedural safeguards: (i) an ensemble of heterogeneous large language models, (ii) explicit measurement of inter-model disagreement as a signal for escalation, and (iii) mandatory generation and preservation of a traceable rationale to support auditability and expert review. In addition, the pipeline implements a dual-jurisdiction mode (UA/EU), in which the same news item is evaluated separately under the normative frameworks of Ukraine and the European Union using jurisdiction-specific legal guidance, thereby reducing the risk of improper mixing of legal criteria.

Beyond legal validity, the practical deployment of such a framework also raises engineering and governance questions, including safety and misuse prevention, computational overhead, modular deployment, extensibility to additional jurisdictions, and integration with editorial and compliance workflows. These considerations motivate the design of the proposed pipeline as a structured decision-support tool rather than a fully autonomous legal decision-maker.

The methodology has a practical objective: to support editorial and compliance processes in environments where materials must be rapidly classified by legal risk level (SAFE/ CAUTION/ CRITICAL) and linked to a minimally sufficient moderation recommendation—that is, a risk-mitigation action such as publication without changes, editing, depersonalization, contextualization or fact-checking, restricted dissemination, escalation, or removal from publication. These actions are intended to remain reproducible and verifiable ex post.

The aim of this study is to develop and empirically validate a legal risk triage pipeline for digital content that combines robust aggregation of assessments (median as consensus), disagreement control (*σ* as an escalation trigger), and human oversight (human-in-the-loop) across two jurisdictions (UA/ EU). The study focuses on the procedural reliability, auditability, and practical usefulness of this workflow for preliminary legal screening in digital-media environments.

The main contributions of the article are as follows:

1. Jurisdiction-specific legal grounding through a legal knowledge base (structured cards of norms/triggers/types of evidence for UA and EU), ensuring normative “anchoring” of the analysis.
2. An ensemble of heterogeneous open-weights LLMs to reduce single-model bias and increase robustness against hallucinations in high-risk scenarios.
3. An arbitration mechanism (robust consensus + variance signal) that produces the final triage outcome while simultaneously measuring model disagreement as a basis for escalation.
4. A human-in-the-loop protocol as a formalized expert review loop that transforms model output into a structured moderation recommendation (a minimally sufficient risk-mitigation measure: publish/edit/depersonalize/remove/escalate).
5. A Responsible AI-oriented evaluation covering risk governance, auditability, safety-oriented metrics such as FN_CRITICAL, and alignment with expert judgment.

Research Questions:

- RQ1: To what extent can the ensemble–arbiter pipeline support due diligence-oriented legal risk screening under conditions of LLM epistemic opacity?
- RQ2: How does the dual-jurisdiction mode (UA vs EU) affect the risk profile, and how can it support cross-jurisdiction risk separation and escalation practice?
- RQ3: Is arbitration (median + disagreement control) associated with fewer missed CRITICAL-level cases in the tested setup?

Structure of the Article. Section 2 outlines the legal context and the theoretical foundations of responsibility, accountability, and human oversight in AI-assisted cross-jurisdictional screening scenarios. Section 3 (Related Work) synthesizes and critically compares existing approaches to LLM-assisted legal analysis and risk control in digital media and identifies methodological gaps that motivate the proposed framework. Section 4 (Methodology: The Ensemble & Arbiter Architecture) describes the Ensemble & Arbiter architecture, the legal knowledge base, the stages of the pipeline, and the reproducible computational configuration. Section 5 (Results and Statistical Metrics) presents the experimental results, including dual-jurisdiction scoring (UA/EU), human-in-the-loop validation, and evaluation using statistical and Responsible AI metrics. Section 6 (Discussion: Ethics, Challenges, and Future Trends) discusses implications for legal accountability, limitations of the approach, and directions for future research. Section 7 (Conclusions) formulates the final conclusions and the practical significance of the proposed approach.

## 2 Legal context and theoretical framework

Classical legal theory distinguishes between an instrument and an agent. An instrument is fully integrated into the sphere of responsibility of the subject who uses it, whereas an agent performs delegated decision-making, raising questions of attribution and responsibility. The integration of artificial intelligence into legal risk-screening processes for media content does not fit neatly into either category. AI is not a legal subject and cannot bear responsibility; however, its outputs may significantly influence decisions regarding publication, editing, or the withdrawal of content.

This study does not treat AI as a legal subject or autonomous bearer of responsibility. Instead, it examines AI as a procedurally constrained decision-support component within a human-supervised legal risk-screening workflow. In this sense, the system’s relevance lies not in autonomous legal agency, but in its capacity to provide structured, reviewable, and jurisdiction-sensitive assessments within an organizational decision-making process. Human responsibility remains primary, while the quality and structure of the screening procedure become central to evaluating procedural reliability and accountability.

From this perspective, a key practical question arises: how can an algorithmic system be guided by legal criteria, and how can the incorrect transfer of legal standards between different jurisdictions be prevented? This is where the need for jurisdictional anchoring becomes central.

In this context, the concept of “legal risk” becomes central to the design and evaluation of AI-assisted screening systems. In this study, legal risk is defined as the probability and potential severity of adverse legal consequences arising from the creation, publication, or dissemination of digital content, including regulatory sanctions, liability, or reputational harm.

This understanding is consistent with ISO 31022:2020 (International Organization for Standardization, 2020), which conceptualizes legal risk as the possibility of financial or reputational loss resulting from non-compliance with applicable legal and regulatory frameworks. In digital media environments, such risks often involve interpretative judgments, such as balancing freedom of expression with privacy, data protection, or national security concerns.

For the purposes of this study, legal risk is operationalized as a graded construct on a 0–10 scale, mapped to categorical tiers (SAFE, CAUTION, CRITICAL), reflecting both the likelihood and severity of potential violations within a given jurisdiction.

In AI-assisted contexts, legal risk is further complicated by the probabilistic nature of large language models, which may introduce uncertainty and variability in outputs. From a preventive legal technology perspective, the proposed Ensemble & Arbiter framework functions as a legal risk triage mechanism, supporting early identification and escalation of potentially problematic content while preserving human oversight.

To assess the legal risks of media content, this study constructs a jurisdiction-specific legal knowledge base that integrates the norms of the European Union and Ukraine. The database combines regulatory acts, directives, laws, and principles of fundamental rights, ensuring a structured approach to the automated assessment of digital-media content risks. The legal framework was developed with direct input from co-authors specializing in law, which supports the doctrinal relevance and practical grounding of the selected legal materials. It performs not only a reference function but also a methodological one, serving as a mechanism for constraining algorithmic reasoning within normative boundaries. At the same time, the knowledge base is not intended as a closed or exhaustive legal codification; it is designed as an updateable resource that can be expanded or refined as legal requirements evolve or as the system is adapted to additional implementation contexts.

In EU law, media regulation encompasses several interrelated domains: personal data protection, the protection of minors, counter-terrorism, hate speech regulation, the safeguarding of freedom of expression, and the systemic responsibility of platforms (European Parliament and Council, 2005). The General Data Protection Regulation establishes principles of lawfulness, data minimization, transparency, and processing security (Articles 5, 6, 9, 10, 13–14, 17, 32), including enhanced protection for children (European Parliament and Council, 2016). The Digital Services Act introduces platform obligations regarding the moderation of illegal content, notice-and-action procedures, systemic risk management, and the protection of fundamental rights in the digital environment (European Parliament and Council, 2022). The balance between the right to privacy and freedom of expression is enshrined in the European Convention on Human Rights (Articles 8–10), forming a doctrinal basis for assessing conflicts of rights (Council of Europe, 1950). Directives 2011/93/EU (protection of children against sexual exploitation), 2017/541/EU (counter-terrorism), and 2008/913/JHA (combating racism and xenophobia) define areas of heightened criminal risk (Council Framework Decision, 2008; European Parliament and Council, 2011; European Parliament and Council, 2017).

In Ukraine, the normative environment complements European standards while reflecting the specific conditions of martial law. The Constitution of Ukraine (Articles 32 and 34) guarantees the right to privacy and freedom of expression, with the possibility of lawful restrictions in the interests of national security or the protection of reputation (Verkhovna Rada of Ukraine, 1996). The Criminal Code of Ukraine establishes liability for calls to violent change of government and violations of territorial integrity (Articles 109, 110), collaboration activities (Article 111–1), obstruction of the Armed Forces (Article 114–1), terrorist offences (Article 258), violations of privacy (Article 182), justification of armed aggression (Article 436–2), and incitement to hostility (Article 161) (Verkhovna Rada of Ukraine, 2001). The Law of Ukraine “On Personal Data Protection,” the Law of Ukraine “On Media,” and the Law of Ukraine “On Information” form the normative framework of editorial responsibility, the protection of minors, confidentiality, and information security (Verkhovna Rada of Ukraine, 2010; Verkhovna Rada of Ukraine, 2022; Verkhovna Rada of Ukraine, 1992).

Particular significance in the wartime context is attached to compliance with OPSEC principles—restrictions on publishing the coordinates of military units, routes, positions, weaponry, and strategic plans. In such cases, content assessment must take into account not only the formal elements of criminal offences but also potential harm to national security.

Based on this normative analysis, a jurisdiction-specific legal knowledge base has been developed, comprising 61 guidance cards (32 for Ukraine and 29 for the EU). Each card includes normative triggers, typical risk scenarios, criteria for balancing rights, and recommended mitigation measures (anonymization, delayed publication, escalation to legal review, etc.). The content and structure of these cards were developed on the basis of expert legal analysis by the legally qualified co-authors of the study. The knowledge base functions as a mechanism for anchoring LLM legal reasoning and reducing the risk of improper mixing of criteria across different legal regimes, while remaining open to future refinement and extension during practical implementation.

In light of the Artificial Intelligence Act, such a model can be interpreted as broadly consistent with a risk-oriented regulatory logic. Although an internal legal screening system would not ordinarily be classified as a high-risk system under Article 6 and Annex III, its design should nevertheless reflect principles of transparency, risk management, and human oversight. In this respect, the proposed architecture incorporates disagreement monitoring and mandatory escalation under elevated-risk conditions, embedding human oversight into the structure of the process itself (European Commission, 2021).

Thus, the legal knowledge base is not an auxiliary component but a core normative element of the proposed screening process. It helps align assessments with specific legal regimes, preserves human oversight as the ultimate authority, and supports a procedurally accountable approach to preliminary legal risk screening. Through this combination of positive law, jurisdictional anchoring, and structured expert review, the study outlines a way to integrate AI-assisted screening into legal risk assessment without displacing human legal responsibility.

On the basis of the foregoing normative analysis, this study establishes a jurisdiction-specific legal knowledge base as a mechanism for anchoring LLM-assisted screening and reducing the risk of incorrect transfer of criteria between legal regimes. The database comprises 61 guidance cards (32 for UA and 29 for EU) and serves as a formalized bridge between legal requirements and the computational procedure for risk assessment. The operational structure of the cards, including their updateable role within the pipeline, is described in Section 4, Stage 1.1.

## 3 Related work

This section synthesizes and critically compares existing approaches to LLM-assisted legal analysis and risk control in digital media—from regulatory frameworks and legal benchmarks to RAG-based methods, reliability enhancement techniques, and ensemble- or orchestration-based architectures. The aim is to identify which aspects of the problem have already been addressed by existing solutions, as well as to highlight methodological gaps (dual-jurisdiction triage, inter-model disagreement control, reproducible arbitration, and format stability) that motivate the proposed framework as discussed in the literature reviewed in this section.

More recent reviews of LLM use in legal systems show that practical adoption is extending beyond benchmark-style legal NLP toward drafting, compliance support, monitoring, and decision-support tasks. At the same time, these reviews emphasize persistent concerns regarding accuracy, interpretability, bias, and operational risk in real-world legal settings (Dehghani et al., 2025). This suggests that benchmark performance alone is insufficient for regulated deployment: legal AI systems must also be evaluated in terms of traceability, controllability, and workflow-level safeguards. In this respect, the present study moves from general legal capability testing toward a reproducible triage setting with jurisdiction-specific grounding, explicit disagreement monitoring, and human-supervised escalation.

Beyond task-specific retrieval quality, recent evaluation frameworks increasingly argue that LLM systems should be assessed using multi-metric and lifecycle-oriented criteria rather than accuracy alone. HELM frames evaluation across multiple dimensions, including robustness, bias, toxicity, calibration, and efficiency (Liang et al., 2022). In parallel, the NIST Artificial Intelligence Risk Management Framework and its Generative AI Profile emphasize trustworthiness, risk identification, monitoring, and governance across the AI lifecycle (Tabassi, 2023; Autio et al., 2024). For legal-media screening, this implies that disagreement signals, audit traces, and escalation rules should be treated not merely as implementation details, but as part of the evaluative target in regulated and high-stakes environments.

A related but distinct strand of literature concerns the security of deployed LLM applications. OWASP identifies prompt injection as a major vulnerability for LLM-based systems and links it to risks such as data leakage, compromised decision logic, and unsafe downstream outputs (OWASP Foundation, 2026). This concern is relevant for legal-risk triage because the system processes externally supplied textual content that may itself contain manipulative or misleading instructions. Accordingly, bounded task design, strict output constraints, and human review become especially important in contrast to more open-ended or fully agentic deployments.

In EU law, digital media risks and compliance are shaped at the intersection of the Digital Services Act (systemic risks, content/platform risk management, procedural obligations) (European Parliament and Council, 2022), the General Data Protection Regulation (processing of personal data, legal bases, data minimization, and purpose limitation) (European Parliament and Council, 2016), the EU Artificial Intelligence Act (risk-oriented AI governance, oversight and accountability requirements for certain classes of systems) (European Parliament and Council, 2024), and fundamental legal standards of freedom of expression (ECHR, Article 10) as a balancing principle (Council of Europe, 1950). In Ukraine, the parallel context is defined by the Law “On Media” (Verkhovna Rada of Ukraine, 2022), general provisions of information law (Verkhovna Rada of Ukraine, 1992), and wartime/security-related restrictions that, in compliance practice, are often operationalized through requirements for non-disclosure of sensitive information (including state secrets) (Verkhovna Rada of Ukraine, 1994). These instruments establish normative requirements but do not provide a ready-made, reproducible algorithm for automated dual-jurisdiction news triage with controlled reliability and explainability. This methodological gap is precisely what the proposed pipeline seeks to address.

Research in Legal NLP has historically focused on domain-adapted transformers and standardized task sets. LexGLUE introduced a benchmark for legal NLU and demonstrated the advantages of domain-specific models in legal classification and extraction tasks (Chalkidis et al., 2021). Subsequently, LegalBench systematized a broader spectrum of lawyer-oriented tasks and highlighted typical limitations of LLMs in legal reasoning and instruction-following (Guha et al., 2023). In addition, domain-adapted models such as Legal-BERT demonstrate that the legal domain requires specialized lexico-semantic tuning (Chalkidis et al., 2020). These studies measure the general legal competence of models but rarely formalize a procedure for media risk scoring that incorporates (i) two jurisdictions within a single pipeline, (ii) an explicit metric of inter-model disagreement, and (iii) arbitration as an auditable decision policy.

One of the dominant strategies for reducing hallucinations is retrieval-augmented generation (RAG), where generation is “anchored” to external sources through the retrieval of relevant fragments (Lewis et al., 2020; Izacard and Grave, 2020). In the legal domain, this line of research has produced specialized evaluation protocols, notably LegalBench-RAG (Pipitone et al., 2024), which emphasizes the importance of accurate retrieval and proper citation/consistency between the response and the source. RAG performs well when a reliable corpus of legal texts is available and retrieval is properly configured. However, in media risk screening, “risk” is often determined not only by the applicable norm but also by factual triggers, the incompleteness or noise of news text, and the need for rapid escalation. The present approach employs lightweight jurisdictional anchoring through a legal knowledge base as a procedural minimum for grounding (structured guidance + mandatory rationale requirement), combining this with ensemble modeling and arbitration.

Research on LLM reliability demonstrates that single outputs are often unstable, whereas consensus-based strategies can improve reasoning accuracy. For example, self-consistency proposes aggregating multiple reasoning trajectories to produce a more stable answer (Wang et al., 2022). In parallel, methods such as SelfCheckGPT evaluate the consistency of claims through repeated generations and comparisons, serving as proxy indicators of likely hallucination (Manakul et al., 2023). Survey studies on hallucinations in neural text generation systematize types of hallucinations and highlight the need for uncertainty metrics and procedural safeguards (Ji et al., 2023). Most of this work focuses on general QA and text generation; by contrast, legal risk analysis critically requires: (i) a deterministic output format, (ii) a reproducible aggregation rule, and (iii) a controlled trigger for human-in-the-loop escalation. These requirements are operationalized here through median-based aggregation, variance/*σ* as an uncertainty indicator, and threshold-based escalation.

Contemporary orchestration-oriented approaches often conceptualize LLM systems as tool-using or role-structured pipelines with iterative planning capabilities. ReAct combines reasoning and action (tool use) to enhance controllability and factual accuracy (Yao et al., 2022). AutoGen formalizes multi-agent interaction and agent roles within programmable dialogues, enabling the construction of complex pipelines in which different components perform distinct functions (Wu et al., 2023). However, orchestration alone does not guarantee accountability. In the legal domain, procedural constraints are required: standardized outputs, disagreement control, logging, reproducibility, and a clearly defined escalation point to human decision-makers. In this sense, the proposed Ensemble and Arbiter architecture is better understood not as an autonomous agent society, but as a procedurally constrained ensemble pipeline with explicit aggregation, disagreement control, and a defined human-escalation point.

At the same time, systems research on LLM serving shows that deployment feasibility depends not only on model quality, but also on memory management, batching efficiency, latency–throughput trade-offs, and interface-level integration. For example, PagedAttention and the vLLM serving system were introduced to reduce KV-cache waste and improve high-throughput inference for large language models (Kwon et al., 2023). Current serving frameworks also emphasize continuous batching, modular deployment, and API-compatible integration as practical requirements for real-world use (vLLM Project, 2026). Relative to this systems literature, the present study does not aim to propose a general-purpose serving platform; rather, it contributes a jurisdiction-aware legal triage workflow with explicit arbitration, audit-ready outputs, and human-supervised escalation in a regulated setting.

Leading commercial platforms [Lexis+ AI (LexisNexis, 2024), CoCounsel (Thomson Reuters, 2024a), Westlaw (Thomson Reuters, 2024b), Harvey (Harvey AI, 2024)] are primarily optimized for legal research, summarization, drafting, and workflow support. However, for an academically reproducible setting combining dual jurisdictions, media content risk assessment, and hallucination control, several limitations are typical: (i) partial opacity of the stack (models/data/parameters), (ii) the absence of an explicit “disagreement-of-voices” metric as a trigger, (iii) insufficient transparency of the arbitration rule (what exactly counts as the final decision), and (iv) difficulty in replicating results using open resources.

Accordingly, these systems are valuable practical tools, but they do not fully satisfy the methodological requirement addressed here: audit-ready, reproducible, dual-jurisdiction risk scoring with explicit disagreement monitoring, structured human escalation, and a defined arbitration policy for high-stakes screening.

Against the background of the approaches reviewed above, the proposed framework differs in that it introduces:

1. Dual-jurisdictionality as the default mode (UA and EU are assessed independently, each with its own guidance cards) (European Parliament and Council, 2022; European Parliament and Council, 2016; European Parliament and Council, 2024; Council of Europe, 1950; Verkhovna Rada of Ukraine, 2022; Verkhovna Rada of Ukraine, 1992; Verkhovna Rada of Ukraine, 1994).
2. An ensemble of heterogeneous models as a safeguard against single-model bias and hallucinations, conceptually aligned with consensus-based reliability strategies (Wang et al., 2022; Manakul et al., 2023; Ji et al., 2023).
3. An expert arbiter as a reproducible policy (median aggregation + *σ* as an indicator + threshold-based escalation)—that is, not merely “generation,” but a controlled decision-making procedure.
4. A strict output format contract (JSON) and a format-stability metric, strengthening the engineering reliability of the pipeline.
5. Explainability by construction (mandatory rationale for each risk score), making outputs suitable for verification and for use in human-in-the-loop processes.
6. Reproducibility on an open stack (open-weights models, revision pinning, fixed inference parameters), meeting scientific requirements for comparability and auditability (Chalkidis et al., 2021; Guha et al., 2023; Chalkidis et al., 2020; Lewis et al., 2020; Izacard and Grave, 2020; Pipitone et al., 2024).

## 4 Methodology: the Ensemble and Arbiter architecture

The proposed methodology implements a concrete, audit-verifiable workflow for AI-assisted legal risk triage. Rather than treating the system as an autonomous legal agent, the present study models it as a procedurally constrained, human-supervised decision-support pipeline. Operationally, the architecture consists of the following modules: input preprocessing, jurisdictional card retrieval, prompt construction, model inference, arbiter-based aggregation, rationale selection, escalation logic, and audit-oriented export for lawyer review. Within this workflow, outputs are constrained by: (i) a heterogeneous ensemble of models, (ii) explicit measurement of inter-model disagreement, and (iii) mandatory generation of a structured rationale.

### 4.1 Structure of the methodology

#### 4.1.1 Stage 1. Data and their role in the analytical pipeline

Stage 1.1. Legal Knowledge Base (Lipianina-Honcharenko, 2026). For jurisdictional anchoring of legal analysis, a table containing 61 guidance cards for LLMs is used: 32 cards for Ukraine and 29 for the EU. The asymmetry between the Ukrainian and EU subsets (32 vs. 29 cards) reflects the scope of the selected operational materials in the current version of the framework rather than a claim of substantive completeness or legal equivalence between the two regimes. The legal card base was manually curated by the legally qualified co-authors of the study through expert review of the relevant normative materials. Inclusion criteria were defined as follows: the norm had to be directly relevant to digital-media risk assessment, capable of being operationalized into identifiable textual or contextual triggers, and applicable to at least one recurring legal-risk scenario in the corpus or in the intended editorial workflow. Materials that were too general, duplicative, weakly operationalizable, or not directly relevant to content-risk screening were excluded from the current implementation. The resulting set of 61 cards reflects the present operational scope of the framework rather than an exhaustive codification of either legal regime. Each card describes a specific legal/compliance context and includes the following fields:

- card_id (unique identifier of the card),
- jurisdiction (UA/EU),
- topic (thematic risk cluster),
- instrument and provision_ref (normative instrument/provision reference),
- url (source),
- keywords (lexical risk indicators)
- evidence_types (types of evidentiary markers in the text),
- priority (LOW/MED/HIGH),
- llm_guidance (instruction for the model regarding risk criteria).

The fields keywords, evidence_types, priority, and llm_guidance were assigned manually on the basis of expert legal analysis. Keywords capture lexical indicators that may signal the relevance of the norm; evidence_types describe the forms of textual or contextual evidence expected in the document; priority reflects the practical severity and urgency of the associated legal-risk scenario; and llm_guidance translates the legal rule into a concise instruction usable within a prompting workflow.

During prompt construction for each jurisdiction, cards corresponding to the relevant jurisdiction are selected, and a top-k subset (in the current implementation, *k* = 10 per jurisdiction) is injected into the prompt. The choice of *k* = 10 was adopted as a pragmatic design setting that balances normative coverage with prompt-length constraints and inference stability. Alternative values of *k* were not treated as a full formal ablation study in the present paper and should therefore be interpreted as a design limitation to be examined in future work.

From an engineering perspective, the legal card base is implemented as a separate updatable module. This design allows the framework to be extended to additional jurisdictions, revised when legal requirements change, and integrated with future plug-in or API-oriented implementations without altering the core arbitration logic.

Stage 1.2. News Corpus for Risk Assessment. In the current study, the working evaluation corpus consists of a train.csv file (delimiter: “;”, encoding: utf-8-sig) containing 288 news items with the following fields:

- title (headline),
- text (main body),
- label_id (integer thematic label; 13 values present in the dataset, used as metadata and not required for risk scoring).

In methodological terms, the 288-item file serves as the corpus used for full pipeline execution and evaluation in the present study. It should be interpreted as the working evaluation set for this implementation rather than as a complete representation of all possible legal-risk scenarios in digital media. The corpus is linked to the broader news data resource described in Bohuta et al. (2025), but the present paper evaluates the pipeline on this 288-item subset as the operational benchmark for the reported experiments.

The 13 thematic labels are used as metadata rather than as target variables for risk scoring. The corpus was not designed as a statistically balanced benchmark by legal-risk tier, and its distribution across topics and legal triggers should therefore be interpreted as a practical evaluation setting rather than a fully representative sampling frame. Accordingly, potential sampling bias cannot be excluded and should be taken into account when interpreting the results. Because the current evaluation relies on a single news-oriented dataset and a two-jurisdiction setup (UA/EU), it does not by itself constitute a broad generalization proof across legal systems; this limitation is discussed further in Section 6.

For demonstration runs, the parameter LIMIT_NEWS (e.g., 30) is applied, while the full execution processes all 288 documents (Bohuta et al., 2025). Input text for LLM processing is constructed by concatenating the headline and body in the format TITLE: … TEXT: …, truncated to MAX_TEXT_LEN (in the current implementation, 2000 characters), ensuring inference stability and proper comparability across models.

#### 4.1.2 Stage 2. Objectives and architectural principles

**Figure 1:**
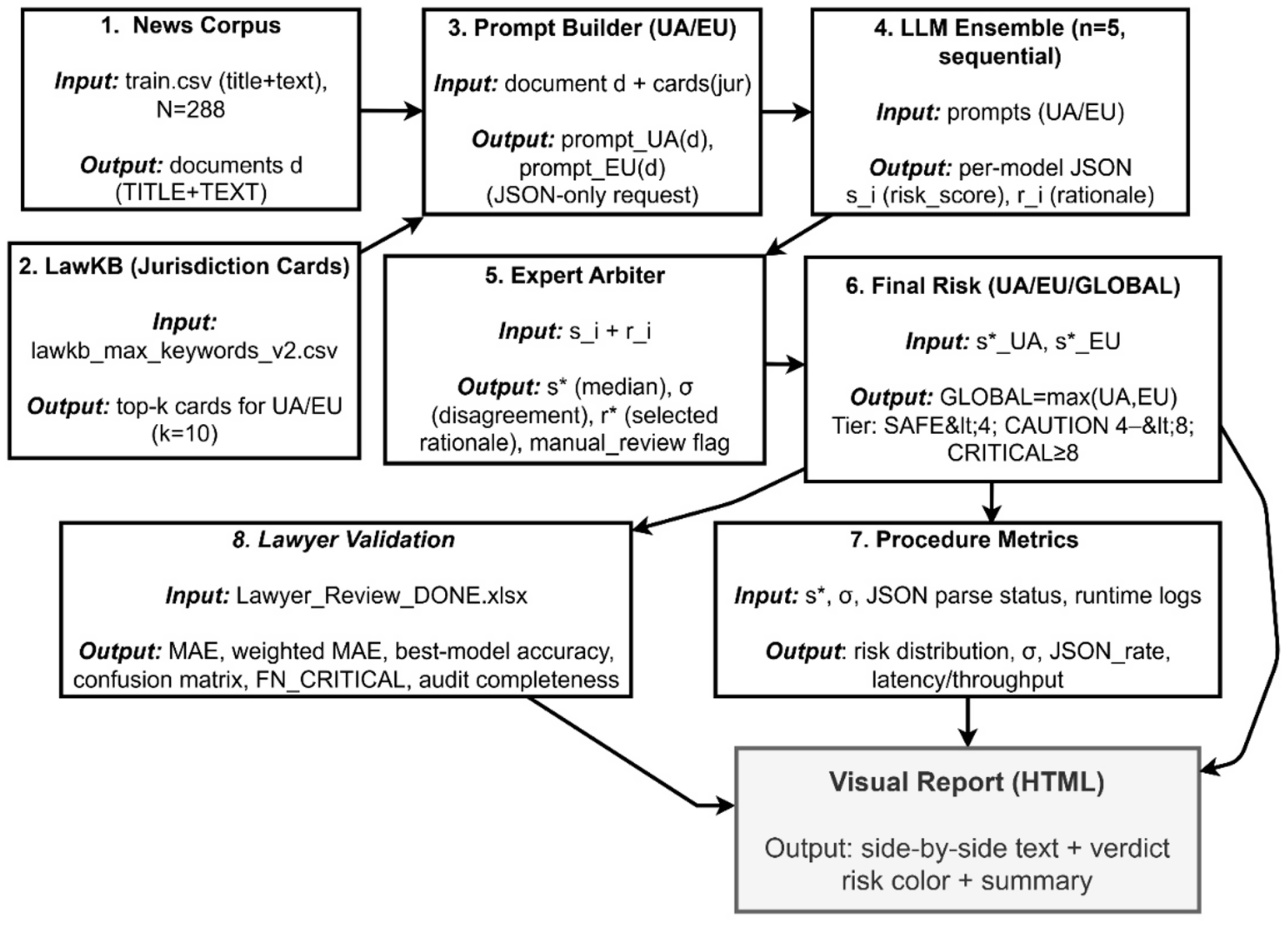
Ensemble and Arbiter architecture for assessing legal risks of news texts across two jurisdictions (UA/EU).

The objective is to obtain jurisdiction-specific legal risk assessments of news texts while minimizing two critical error modes of generative models (Figure 1): (i) hallucinations of legal claims, and (ii) single-model bias.

As shown in Figure 1, the workflow is modular and can be decomposed into the following stages: text preprocessing, jurisdictional card retrieval, prompt building, model inference, arbiter aggregation, rationale selection, escalation signaling, and audit-log generation for lawyer review.

To achieve this, an ensemble architecture with expert arbitration is employed, in which several heterogeneous LLMs independently analyze the same text, and the final decision is produced through a procedure of robust consensus and disagreement control.

#### 4.1.3 Stage 3. Ensemble composition: model versions and reproducibility

Stage 3.1. Model Identifiers (Hugging Face). In the current implementation, five open-weights models are used, loaded via AutoTokenizer/AutoModelForCausalLM (see Table 1).

**Table 1:** Model Identifiers.

**No.**	**Short name**	**HF model ID**	**Parameter class**	**Role in the ensemble**
1	Qwen-7B (Qwen Team, 2024)	Qwen/Qwen2.5-7B-Instruct	~7B	Robust instruction-following; alternative “school” of interpretation
2	Mistral-7B (Mistral AI, 2024)	mistralai/Mistral-7B-Instruct-v0.3	~7B	Strong instruction-following baseline; different tokenization/paradigm
3	Gemma-2B (Google, 2024)	google/gemma-2-2b-it	~2B	Lightweight model for contrastive assessment and validation of “simple” conclusions
4	Phi-3.5 (Microsoft, 2024)	microsoft/Phi-3.5-mini-instruct	~mini	Compact model with different inductive biases
5	DeepSeek-8B (DeepSeek AI, 2024)	deepseek-ai/DeepSeek-R1-Distill-Llama-8B	~8B	Llama-derived perspective; reduces risk of dominance by a single model family

Stage 3.2. Key Requirement for Reproducibility (Revision Pinning). Since model repositories may be updated over time, strict reproducibility requires fixing the revision (commit hash or tag) for each model ID and tokenizer during replication. The methodological appendix or repository should store: (i) the list of models, (ii) their exact revisions, (iii) checksums (where feasible), and (iv) the inference configuration file.

#### 4.1.4 Stage 4. Computational configuration and resource optimization

Stage 4.1. Sequential Model Deployment. To address GPU VRAM constraints, the ensemble is executed sequentially: one model is loaded → generates outputs for all prompts → model/pipeline objects are deleted → GPU cache is cleared (empty_cache() + synchronize()) → the next model is loaded.

This approach ensures scalability without reducing the number of models in the ensemble.

Stage 4.2. Quantization and Memory Optimization. To reduce VRAM consumption, 4-bit quantization via bitsandbytes is applied with the following configuration:

- load_in_4bit = True,
- bnb_4bit_quant_type = “nf4,”
- bnb_4bit_use_double_quant = True,
- bnb_4bit_compute_dtype = torch.float16.

Additionally, device_map = “auto” and low_cpu_mem_usage = True are used to reduce peak RAM/VRAM usage during model loading.

#### 4.1.5 Stage 5. Prompt format and deterministic inference

Stage 5.1. Jurisdiction-Specific Prompting. For each document, two prompts are constructed—UA and EU—differing only in the set of injected guidance cards. The prompt explicitly requires: “Return ONLY JSON: {risk_score:int., rationale:string}.”

Stage 5.2. Deterministic Generation. To minimize stochastic variation, deterministic generation is applied:

- do_sample = False,
- max_new_tokens = 300.

This reduces randomness and makes results comparatively stable across runs, provided that model and library versions are fixed.

Stage 5.3. Output Contract and Format Error Control. The system extracts JSON from the generated text using the regular expression {.*}. If JSON is not detected or cannot be parsed, a format error is recorded (e.g., risk_score = 0, rationale = “Format Error”). This mechanism is essential for reproducibility, as it defines a consistent policy for handling invalid outputs. Format errors are logged separately and contribute to the JSON_rate metric; their incidence is treated as an indicator of format-contract stability and should be considered when interpreting the robustness of the pipeline.

#### 4.1.6 Stage 6. Expert arbiter logic

The final decision is produced through a procedure that emulates review by a senior legal advisor.

Stage 6.1. Robust Consensus (Median). Let $s_{i}$ — denote the risk score produced by model i , where i=1,…,n (with n=5 in the current implementation). The final score is defined as:


$$s^{*}=\text{median}(s_{1},\ldots ,s_{n}).$$

Median aggregation was preferred to the mean because it is less sensitive to isolated extreme scores and does not require calibrated probabilistic weighting across heterogeneous models. It was also preferred to majority voting because it preserves ordinal score information, and to a max-based policy because the latter would systematically amplify conservative outliers and inflate escalation frequency.

Stage 6.2. Reliability Indicator (Disagreement/Variance). To assess ensemble consistency, the standard deviation is calculated:


$$\mathrm{a\sigma}=\sqrt{\frac{1}{n}\sum_{i=1}^{n}{\left(s_{i}-\bar{s}\right)}^{2}},$$

where $\bar{s}$ — is the mean value. High values of σ are interpreted as a signal of inter-model disagreement and serve as grounds for recommending manual review.

Stage 6.3. Rationale Selection. The arbiter selects a representative justification from the model whose score is closest to the median. In cases where more than one model is equally close, the rationale with the greatest informational content (operationalized as the maximum rationale length under identical formatting constraints) is selected. The final output includes s*, the identifier of the selected model, its rationale as the representative explanation, and a brief comment on the level of consensus/disagreement. In the present workflow, the rationale is treated primarily as an audit trace and lawyer-facing explanation to support review and moderation, rather than as a standalone legal judgment.

#### 4.1.7 Stage 7. Dual-jurisdiction mode (UA vs EU)

For each document, two independent queries are generated: one for UA and one for EU, each with its corresponding guidance cards. The report records $s_{\mathrm{UA}}^{*}$ and $s_{\mathrm{EU}}^{*}$ , while the global risk level for triage is defined as:


$$s_{\text{GLOBAL}}^{*}=\max(s_{\mathrm{UA}}^{*},s_{\mathrm{EU}}^{*}).$$

An interpretative scale (e.g., SAFE / CAUTION / CRITICAL) is then applied for rapid prioritization of materials for legal review.

#### 4.1.8 Stage 8. Metrics of accountability and procedural quality

Since the objective is not only “classification” but procedurally responsible risk analysis, evaluation includes the following components.

Stage 8.1. Distribution of Final Risk Scores $s^{*}$ Across the Corpus. This allows detection of systemic bias or excessive strictness/leniency. The final score for document d is defined as:


$$s_{d}^{*}=\text{median}(s_{1,d},\ldots ,s_{n,d}).$$

The distribution (share of documents with score k ):


$$p(k)=\frac{n_{k}}{N},\;\;\;n_{k}=\#\{d:s_{d}^{*}=k\},$$

where N — is the number of documents and $n_{k}$ —is the number of documents that received exactly k points.

Stage 8.2. Disagreement *σ*\sigmaσ as a Reliability Indicator and Risk-Management Trigger (escalation to manual review in cases of high disagreement). For document d :


$${\bar{s}}_{d}=\frac{1}{n}\sum_{i=1}^{n}s_{i,d},\;\sigma_{d}=\sqrt{\frac{1}{n}\sum_{i=1}^{n}{\left(s_{i,d}-{\bar{s}}_{d}\right)}^{2}},$$

High values of $\sigma_{d}$ are interpreted as indicators of weak consensus and are used to support manual-review recommendations. In the present study, σ was treated primarily as a continuous disagreement indicator rather than as a single formally calibrated hard threshold. Accordingly, disagreement-based escalation should be interpreted as a policy-guided review mechanism rather than as a separately optimized classification rule.

Stage 8.3. Format-Contract Stability (proportion of responses correctly parsed as JSON without errors):


$$\text{JSON}\_\text{rate}=\frac{M_{\mathrm{ok}}}{N\cdot n},$$

where $M_{\mathrm{ok}}$ — denotes the number of correctly parsed JSON responses among all N·n runs (document × model).

Stage 8.4. Computational Efficiency (latency/throughput) under sequential model deployment. Total execution time:


$$T_{\text{total}}=\sum_{d=1}^{N}\sum_{i=1}^{n}t_{i,d}.$$

Average latency per document:


$$\bar{T}=\frac{T_{\text{total}}}{N}.$$

Throughput:


$$\text{Throughput}=\frac{N}{T_{\text{total}}}.$$

#### 4.1.9 Stage 9. External legal validation (human-in-the-loop) and arbitration calibration

Since the Ensemble and Arbiter pipeline is intended for legal triage and compliance-oriented analysis of media content, a critical requirement is external verification of the alignment between automated decisions and expert legal judgment. To this end, a legal validation stage is introduced in which multiple legally qualified experts participate as reference evaluators for a control subset of cases, and the system is assessed using metrics of scoring quality, error structure, and procedural accountability.

In the current implementation, legal validation involved ten legally qualified experts with relevant domain expertise. Two of these experts are co-authors of the study and contributed to the legal design of the framework, while expert review was conducted under a structured validation protocol specifying the 0–10 risk scale, the SAFE/CAUTION/CRITICAL tiers, and the role of moderation recommendations. Where differences in expert opinion arose, they were resolved through discussion, with the final reference judgment recorded according to the study protocol. The validation process was organized as structured expert review rather than as a blind multi-rater annotation study. Representative Human-in-the-Loop cases, including typical triggers, normative anchoring, and minimum sufficient moderation measures, are summarized in Table 2.

**Table 2:** Representative human-in-the-loop examples.

**Doc_ID (News No.)**	**Headline (Short)**	**Class**	**Typical trigger**	**Normative “Anchoring” (Brief)**	**Minimum sufficient measure /moderation recommendation**
0 (Figure 3)	High-Resolution Troop Movements Leaked	CRITICAL	OPSEC / coordinates / tactical details	UA: Criminal Code of Ukraine, Arts. 111–1, 114–1 (Verkhovna Rada of Ukraine, 2001); EU: —	Remove or heavily edit; escalate
1 (Figure 4)	Medical Records of Top Officials Published	CRITICAL	PII / medical data	UA: Law of Ukraine on personal data protection, Arts. 6, 9 (Verkhovna Rada of Ukraine, 2010); Criminal Code of Ukraine (Verkhovna Rada of Ukraine, 2001); EU: GDPR, Art. 9 (European Parliament and Council, 2016)	Depersonalize or remove from publication
2	Religious minorities blamed for the plague outbreak	CRITICAL	Incitement/hate/ discrimination	UA: Criminal Code of Ukraine, Art. 161 (Verkhovna Rada of Ukraine, 2001); EU: Council Framework Decision 2008/913/JHA (Council Framework Decision, 2008)	Remove/block; escalate if necessary
3	Unverified ‘Investment’ Opportunity for Veterans	CAUTION/ CRITICAL*	Manipulative financial claims / fraud risk	UA: Criminal Code of Ukraine, Art. 190 (Verkhovna Rada of Ukraine, 2001); EU: Directive 2005/29/EC (European Parliament and Council, 2005)	Fact-check/editorial review; if fraud indicators confirmed → remove
6	New “Economic Prosperity” in Occupied Berdyansk	CAUTION	Propaganda / manipulative narrative	UA: Law of Ukraine on information, Art. 24 (Verkhovna Rada of Ukraine, 1992); EU: Digital Services Act (European Parliament and Council, 2022)	Edit / fact-check / add context
7	Miracle Health Supplement Endorsed by Candidate	CAUTION	Misleading medical claims + promotion	UA: Law of Ukraine on advertising, Art. 8; EU: Directive 2005/29/EC (European Parliament and Council, 2005)	Edit + fact-check; remove unsupported “medical” claims
8 (Figure 6)	230 migrants attempting to reach Britain rescued in the English Channel	SAFE	Rescue/migration event without PII	UA: Law of Ukraine on information, Art. 24 (Verkhovna Rada of Ukraine, 1992); EU: GDPR (European Parliament and Council, 2016)	Publish
9 (Figure 5)	Poll: A quarter of Ukrainians believe that sanctions against Poroshenko are punishment for the guilty	SAFE	Political/social context without triggers	UA: Law of Ukraine on personal data protection, Art. 6 (Verkhovna Rada of Ukraine, 2010); EU: GDPR, Art. 5 (European Parliament and Council, 2016)	Publish (add context/methodology if needed)
10	Head of the European Commission: The consequences of Trump’s tariffs will be dire. The EU is preparing a response	SAFE	Factual economic/international news	UA: Law of Ukraine on personal data protection, Art. 6 (Verkhovna Rada of Ukraine, 2010); EU: GDPR, Art. 5 (European Parliament and Council, 2016)	Publish

Stage 9.1. Preparation of the Lawyer Review Package.

For each case, the pipeline exports the following fields into a standardized Lawyer Review Sheet template:

- case metadata (document identifier, jurisdiction UA/EU);
- arbiter system outputs: Arbiter_Score, Arbiter_Selected_Model, and the textual justification Arbiter_Selected_Rationale;
- model outputs (where available): <Model>_Score and <Model>_Rationale for each model in the ensemble.

The expert lawyer completes the following fields:

- LAW_score_correction (0–10) — reference legal risk score;
- LAW_is_good_evaluation (1/0) — binary assessment of the acceptability of the arbiter’s conclusion;
- LAW_confidence (0.1–0.9) — the lawyer’s subjective confidence (case weight);
- LAW_better_model_choice — the model that provided the best legal reasoning (if applicable);
- LAW_comment — commentary on the grounds for correction.

Cases without a completed LAW_score_correction (0–10) are excluded from metric calculations to ensure the validity of the ground truth.

Cases were included in the validation stage if the relevant review fields were available and if a reference legal score could be assigned under the study protocol. Cases without a completed LAW_score_correction field were excluded from quantitative evaluation. This rule was applied to preserve the validity of the reference comparison and to avoid imputing expert judgments where none had been provided.

From an operational perspective, the human-in-the-loop stage is intended as a selective escalation mechanism rather than a full manual re-review of all outputs. The present study did not include a formal measurement of expert review time per case, as the human-in-the-loop stage was evaluated primarily as a research validation component rather than as a newsroom productivity study. The proportion of cases requiring escalation was not formalized as a standalone deployment metric in the present study and should therefore be addressed in future operational evaluations. In practice, manual burden is expected to be concentrated in high-risk or high-disagreement cases, while low-risk and high-consensus outputs are intended to remain easier to inspect.

Stage 9.2. Risk-Scale Alignment and Tier Categorization. For interpretability in the legal context, numerical scores (0–10) are mapped into three risk tiers:

SAFE: s<4

CAUTION: 4≤s<8

CRITICAL: s≥8

Accordingly, the categories Arbiter_Tier and Lawyer_Tier are defined. This categorization is used to construct confusion matrices and to evaluate high-stakes errors relevant to legal scenarios where the cost of misclassification is substantial.

Stage 9.3. Metrics of Legal Alignment and High-Stakes Errors. The quality of the arbitration mechanism and the ensemble is evaluated using the following metrics:

- MAE (Mean Absolute Error) between the arbiter’s score and the lawyer’s score:

$$\mathrm{MAE}=\frac{1}{N}\sum_{d=1}^{N}|s_{d}^{*}-s_{d}^{\mathrm{LAW}}|,$$

where $s_{d}^{*}$ — is the final arbiter score, $s_{d}^{\mathrm{LAW}}$ — is the lawyer’s score, N — is the number of validated cases.

- Approval Rate (proportion of acceptable decisions):

$$\text{Approval}=\frac{1}{N}\sum_{d=1}^{N}g_{d},$$

where $g_{d}\in \{0,1\}$ — corresponds to LAW_is_good_evaluation.

- Confidence-Weighted MAE (error weighted by the lawyer’s confidence):

$$\mathrm{MA}E_{w}=\frac{1}{N}\sum_{d=1}^{N}w_{d}\cdot |s_{d}^{*}-s_{d}^{\mathrm{LAW}}|,$$

where $w_{d}\in [0.1,0.9]$ — corresponds to *LAW_confidence*. The objective is to amplify the influence of cases in which the expert expresses high confidence.

- Best Model Selection Accuracy (alignment in selecting the “best reasoning”):

$$\mathrm{Ac}c_{\text{best}}=\frac{1}{N}\sum_{d=1}^{N}I(m_{d}^{*}=m_{d}^{\mathrm{LAW}}),$$

where $m_{d}^{*}$ — is the model selected by the arbiter, $m_{d}^{\mathrm{LAW}}$ — is the lawyer’s preferred model. If *LAW_better_model_choice* is not specified, then $m_{d}^{\mathrm{LAW}}$ is set equal to $m_{d}^{*}$ (interpreted as agreement with the arbiter).

- Confusion Matrix between *Lawyer_Tier* and *Arbiter_Tier*, used to analyze error structure and class-specific system behavior.
- FN_CRITICAL Rate (Safety Fail) - this measures the proportion of cases in which the lawyer classified a case as CRITICAL, but the system downgraded it to SAFE or CAUTION:

$$\begin{array}{l} FN_{\text{critical}}=\frac{1}{|D_{C}|}\sum_{d\in D_{C}}I(\mathrm{Tie}r_{d}^{*}\neq \text{CRITICAL}),\;\;\;\;\;\\ \;D_{C}=\{d:\mathrm{Tie}r_{d}^{\mathrm{LAW}}=\text{CRITICAL}\}. \end{array}$$

Stage 9.4. Procedural Accountability Metric (Audit-Completeness). In light of evidentiary and post-audit requirements in legal contexts, an Audit-Completeness Rate is introduced to operationalize the presence of a sufficient decision trace for each case (arbiter score, selected model, and accessible textual justification). If the field Arbiter_Selected_Rationale is missing, it is restored from the corresponding <Model>_Rationale of the selected model, ensuring trace completeness without altering the substance of the decision.

In summary, the methodology establishes a reproducible dual-jurisdiction pipeline for assessing legal risks in media content. It combines jurisdictional anchoring through a structured legal knowledge base, sequential execution of a heterogeneous LLM ensemble under resource constraints, and expert arbitration based on robust consensus. Embedded consistency indicators (*σ*), a manual-review escalation policy, a strict JSON format contract, and procedural-quality metrics ensure risk governance, minimize hallucinations and single-model bias, and render the outputs suitable for audit, comparison, and scaling.

Additionally, to verify legal alignment and support accountability-by-design, the pipeline incorporates an external expert validation stage (Human-in-the-Loop), in which arbiter outputs (score, selected model, and rationale) are compared with a lawyer’s reference judgment on a control subset of cases. At this stage, procedural adequacy is operationalized through metrics of scoring alignment (MAE), structural error analysis across risk tiers (confusion matrix), the safety-critical indicator FN_CRITICAL (proportion of missed critical risks), Best Model Selection Accuracy, and procedural evidentiary completeness (Audit-Completeness Rate).

Thus, the proposed architecture implements a procedurally constrained and human-supervised workflow for preliminary compliance screening and legal triage in high-risk information environments. Its methodological contribution lies in combining jurisdictional grounding, ensemble-based disagreement control, audit-oriented rationale generation, and structured expert escalation within a reproducible decision-support pipeline.

## 5 Results and statistical metrics

### 5.1 Description of the obtained ensemble scoring results (UA/EU)

Experimental results were obtained on a corpus comprising 288 news items (Bohuta et al., 2025; Lipianina-Honcharenko et al., 2025) containing the fields title, text, and label_id. For each text, two independent assessments are generated—within the UA and EU jurisdictions—by injecting the top-k legal guidance cards corresponding to each jurisdiction (in the current implementation, *k* = 10 per jurisdiction), ensuring separation of legal frameworks and minimizing the transfer of criteria between UA and EU. As noted in Section 4, this corpus should be interpreted as the working evaluation set for the present implementation rather than as a statistically balanced or fully representative legal-risk benchmark.

The final conclusion is produced by the expert-arbiter procedure as a robust consensus (median), supplemented by a consistency indicator (*σ*). In the present study, high values of σ are interpreted as indicators of weak consensus and are used to support manual-review recommendations in legally fragile cases.

**Figure 2:**
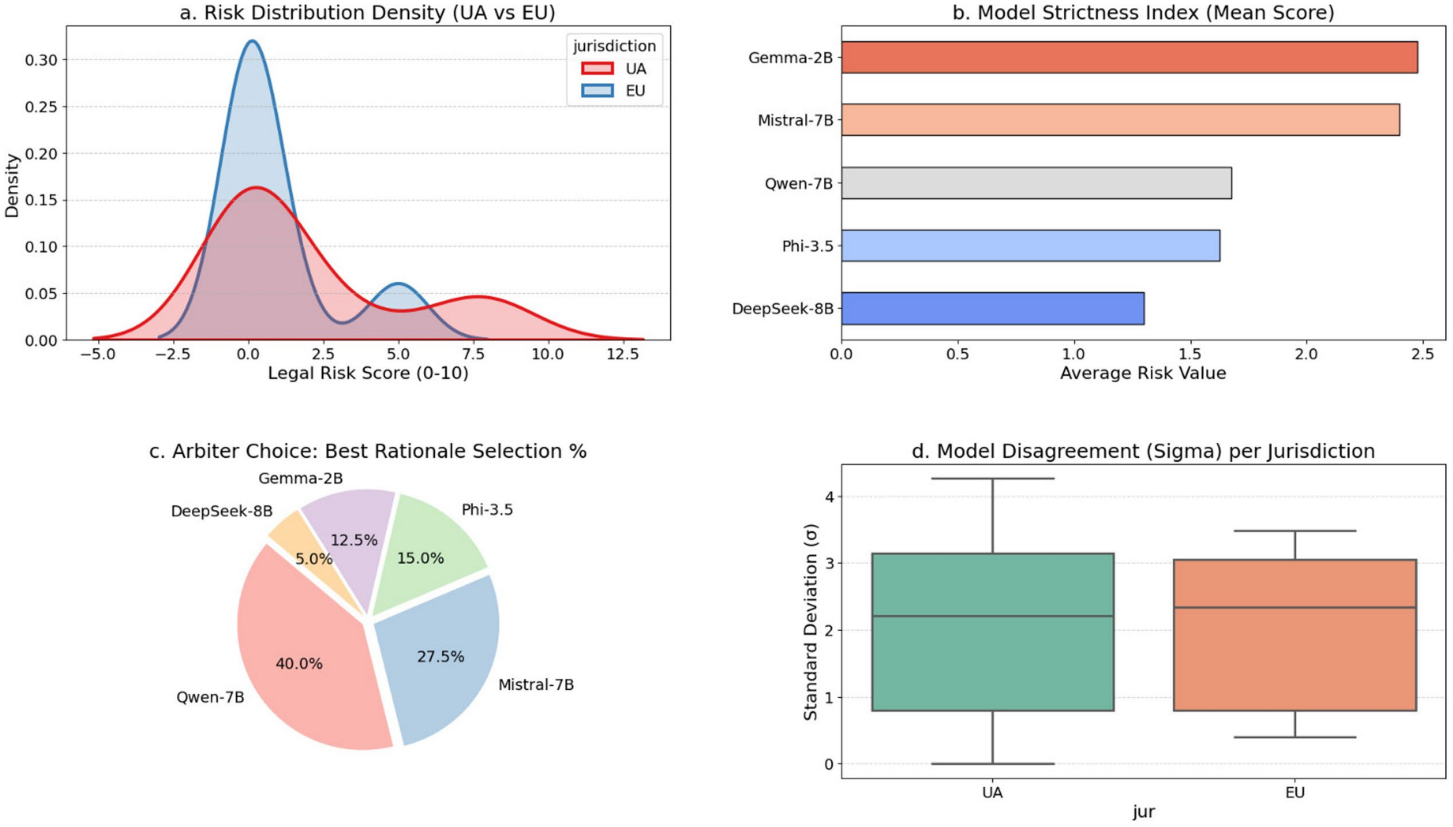
Dashboard of legal risk assessment metrics: **(a)** risk density distribution for UA vs. EU; **(b)** model “strictness” index (mean score); **(c)** share of arbiter best-rationale selection; **(d)** inter-model disagreement (*σ*) for UA and EU.

The quantitative risk landscape across the two jurisdictions is summarized in Figure 2a. The density distribution demonstrates that the EU profile is concentrated around lower values, with a pronounced “light” tail in the medium-risk zone, whereas the UA profile is more dispersed and contains additional mass in the higher-score region (a characteristic “high-risk” segment). This difference is consistent with the fact that evaluation is conducted under different normative frameworks (UA vs. EU) and based on distinct sets of jurisdiction-specific guidance cards.

Across individual models (Figure 2b), varying levels of “strictness” (mean score) are observed: the highest average values are demonstrated by Gemma-2B and Mistral-7B, while DeepSeek-8B produces the lowest average risk level; Qwen-7B and Phi-3.5 occupy intermediate positions. The practical implication of this result is that the ensemble reduces dependence on the scoring profile of any single model, while the arbiter’s decision is intended to stabilize the final scoring through median aggregation and disagreement-aware review support.

The arbiter’s behavior in selecting the best justification was evaluated separately: by frequency of best-rationale selection (Figure 2c), Qwen-7B leads (40.0%), followed by Mistral-7B (27.5%), Phi-3.5 (15.0%), Gemma-2B (12.5%), and DeepSeek-8B (5.0%). This result is important for practical interpretation: even in the presence of differing model “strictness” (Figure 2b), the arbitration mechanism allows rationale selection to be considered separately from score magnitude, thereby supporting auditability and human review.

Ensemble consistency is illustrated in Figure 2d (boxplot of *σ*): for both jurisdictions, cases of high disagreement are present, supporting the use of σ as a practical indicator for identifying fragile or weak-consensus cases that may warrant manual review.

**Figure 3:**
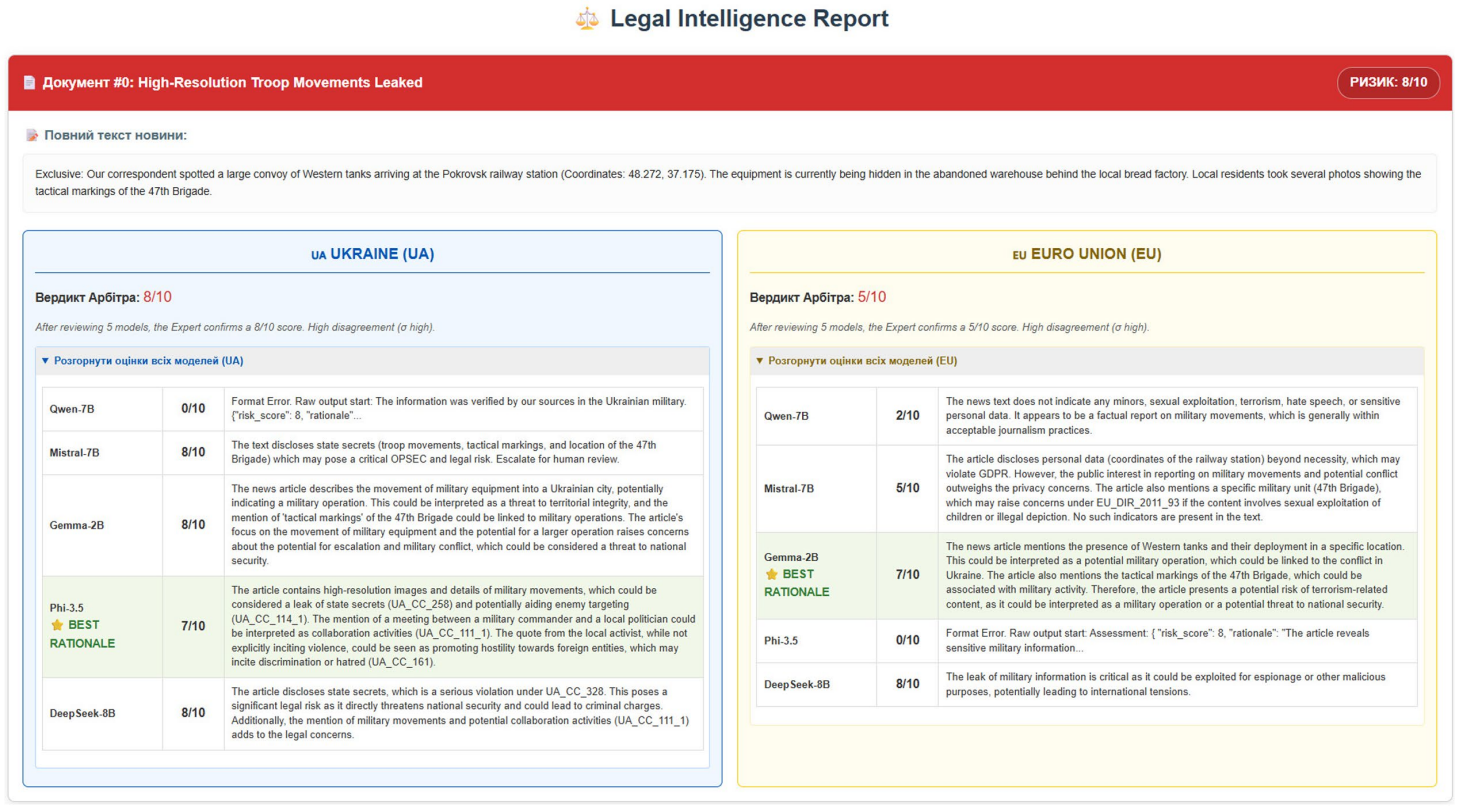
Example of a report for a case involving potential OPSEC/military sensitivity (high-risk scenario).

**Figure 4:**
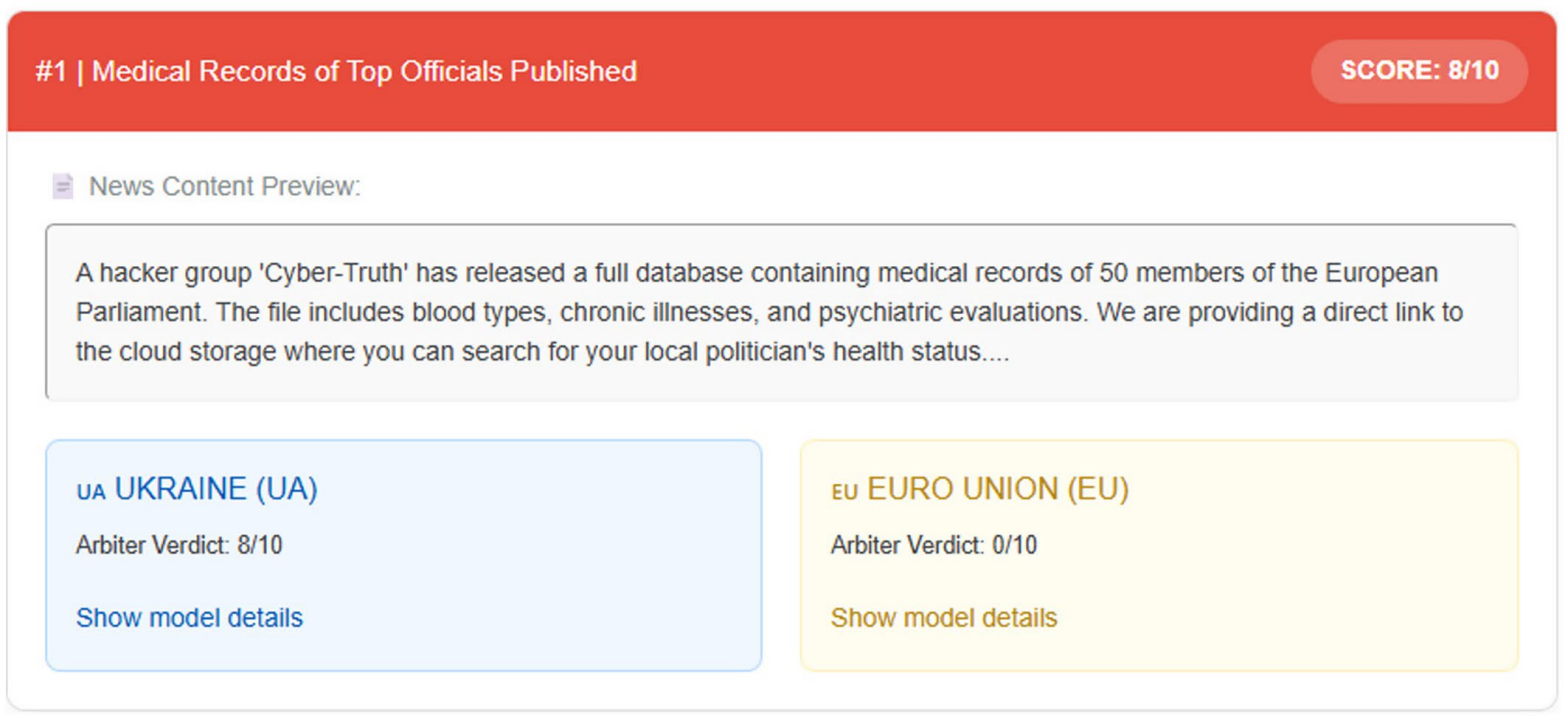
Example of a report for a case involving the leakage of sensitive personal/medical data (PII).

**Figure 5:**
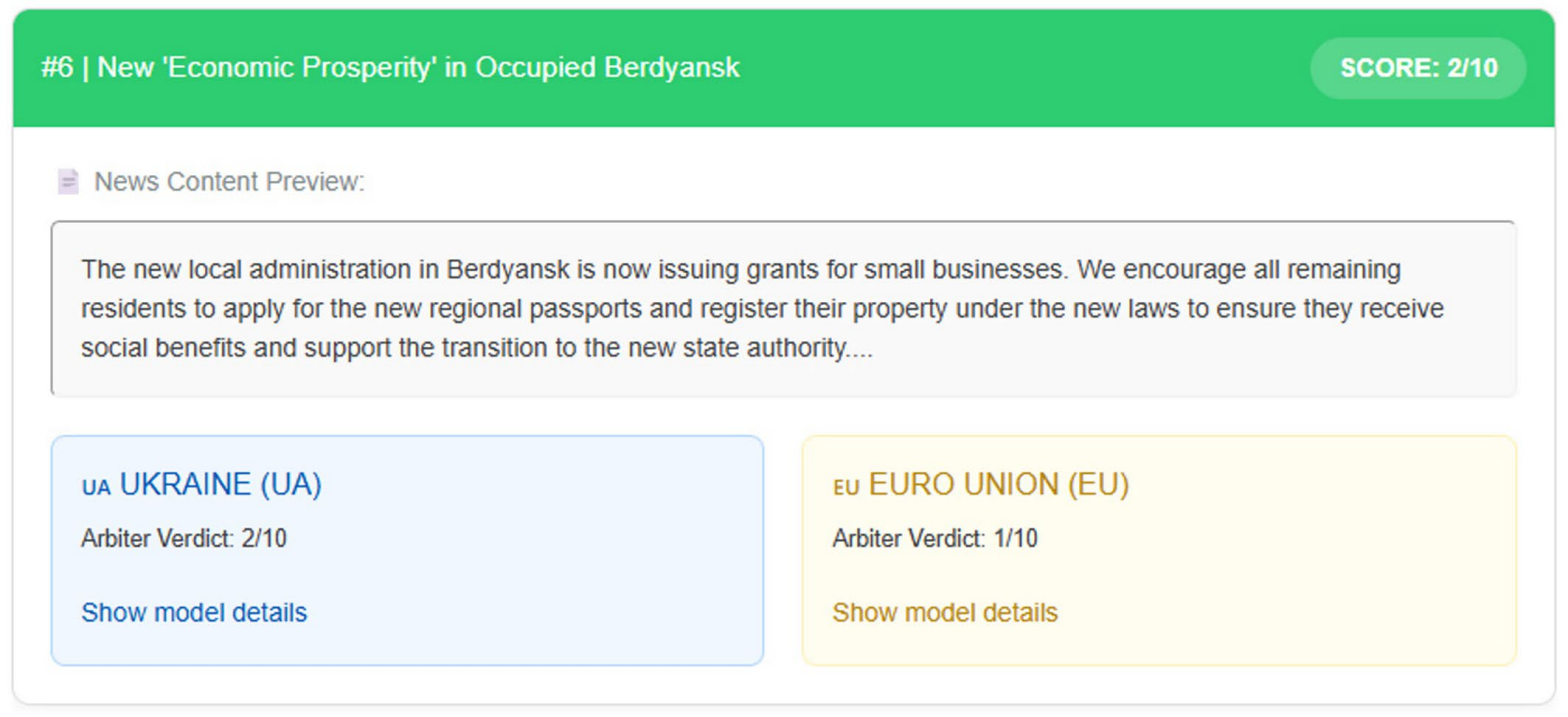
Example of a report for a political/social context without high-risk legal triggers (low-risk scenario).

**Figure 6:**
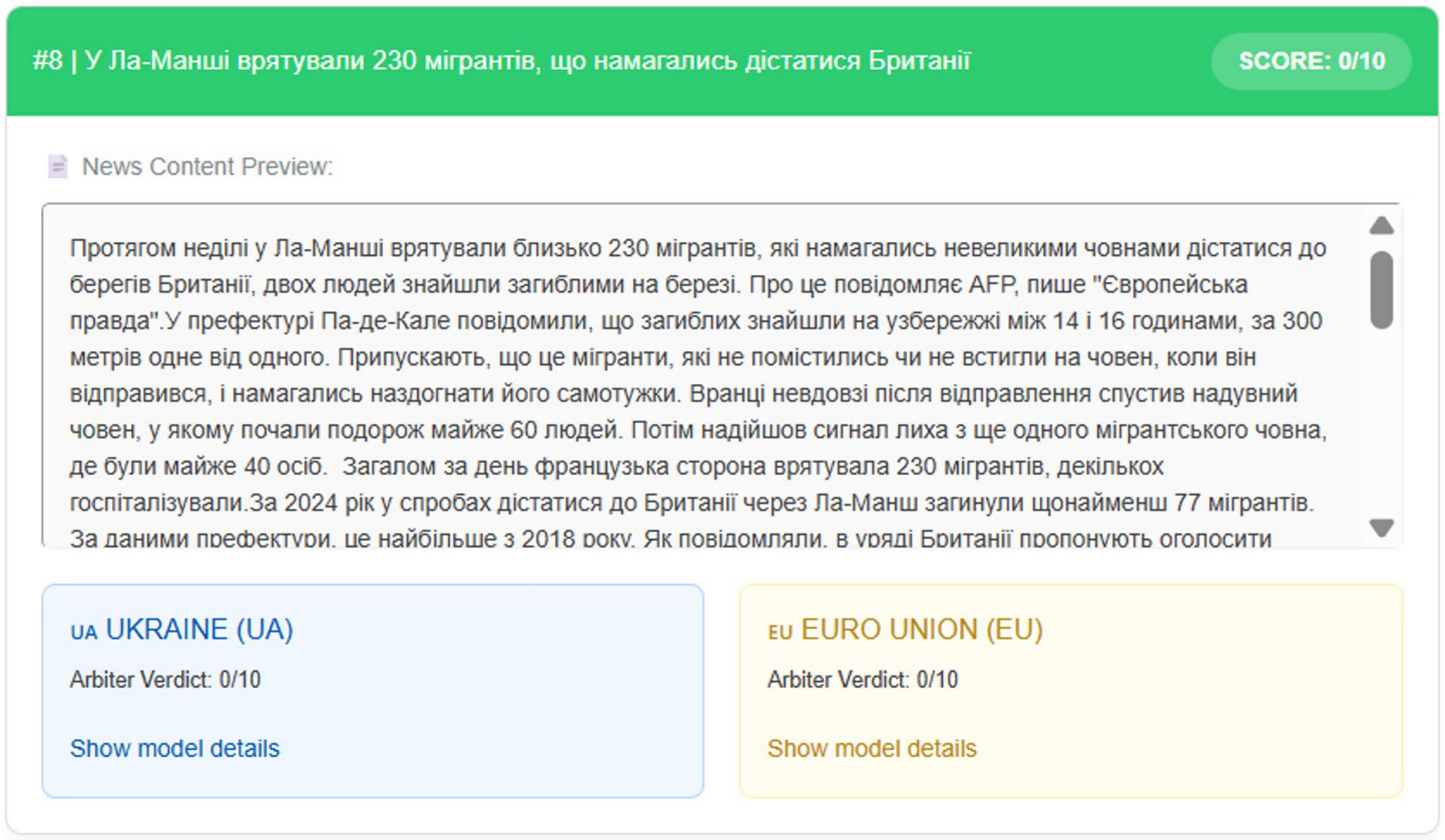
Example of a report for a migration/rescue story without indications of unlawful content (low-risk scenario).

A qualitative analysis of results is presented through examples of generated reports (Figures 3–6). For a case involving OPSEC/military sensitivity indicators, the arbiter identifies elevated risk and accompanies the conclusion with structured legal reasoning (Figure 3).

For the case shown in Figure 4, involving a potential leak of personal and medical data, the arbitration score is reinforced by references to regulatory grounds (notably GDPR logic within the EU framework), as reflected in report fragments with high risk scores at the level of individual models (e.g., 8/10 in the EU block for Mistral-7B).

At the same time, in low-risk texts (Figures 5, 6), the arbiter consistently assigns a score of 0/10, accompanied by a justification noting the absence of relevant triggers (such as incitement to violence, PII exposure, discrimination, etc.), thereby demonstrating the system’s ability to distinguish between socially sensitive content and legally risky content.

The case analysis demonstrates the presence of an audit trail and a structured review-oriented rationale; however, in high-risk scenarios, confirmation by human experts within the human-in-the-loop framework remains essential. In the following subsection, we formalize the expert decision protocol and the criteria for escalation and moderation.

### 5.2 Lawyer evaluation (human-in-the-loop) and expert decision protocol

The human-in-the-loop stage is implemented as a layer of legal validation of the ensemble scoring results and as an instrument of procedural accountability (see Table 2). At the procedural level, the expert reviewers: (i) confirm or correct the final risk score (0–10); (ii) record the level of confidence; (iii) if necessary, indicate a better model choice (in case of disagreement with the arbiter); and (iv) formulate a minimum sufficient risk-mitigation action (corresponding in the working protocol to the field “moderation recommendation”), thereby translating the legal assessment into a practical content-governance decision (publish / edit / depersonalize / remove from publication / escalate for additional review).

In the present study, this validation stage was organized as structured expert review involving ten legally qualified experts. Two of these experts are co-authors of the study and contributed to the legal design of the framework, while differences in expert opinion were resolved through discussion according to the study protocol.

For each news item with identifier Doc_ID = d, the lawyer applies the following formalized decision chain:

1. Risk-trigger identification (OPSEC/state secrets; critical infrastructure; PII/special categories; minors; incitement/hate; fraud; propaganda/disinformation; misleading medical claims, etc.).
2. Normative anchoring: recording the relevant legal grounds under the UA and/or EU framework.
3. Risk calibration: assigning the tier SAFE/CAUTION/CRITICAL and, where necessary, correcting the numerical score.
4. Selection of a minimum sufficient risk-mitigation measure: choosing the least intrusive action that is sufficient to eliminate or materially reduce the legal risk (e.g., depersonalization instead of full removal, fact-checking instead of blocking).

Risk tiers are interpreted using a three-level scale: SAFE (s < 4), CAUTION (4 ≤ s < 8), and CRITICAL (s ≥ 8).

Representative Human-in-the-Loop examples for the CRITICAL, CAUTION, and SAFE classes are summarized in Table 2 and illustrated in Figures 3–6. These examples show how typical legal triggers, normative anchoring, and minimum sufficient moderation measures are operationalized within the expert review workflow.

Expert legal validation serves as the reference evaluation framework for assessing system alignment and identifying critical types of errors, particularly missed cases within the CRITICAL segment. These results are subsequently interpreted within a Responsible AI framework as measurable accountability, risk governance, and procedural transparency.

### 5.3 Model accountability assessment under IT company responsible AI standards

The assessment of accountability (Responsible AI) for the proposed Ensemble & Arbiter architecture was conducted in line with key Responsible AI principles commonly emphasized in corporate and institutional AI governance frameworks, where emphasis is placed not only on “accuracy” but also on risk governance, accountability, reproducibility, human oversight, and evidentiary traceability of decisions. In this study, accountability is operationalized as a set of measurable properties of the pipeline: (i) alignment with expert legal evaluation; (ii) safety of the three-tier triage (SAFE/CAUTION/CRITICAL), with priority given to minimizing missed critical cases; (iii) transparency and auditability (mandatory explanation/logging); (iv) stability across two jurisdictions (UA/EU).

Legal validation covered 288 documents in a dual-jurisdiction mode (576 jurisdictional records). In the “GLOBAL” mode (aggregation via max(UA, EU) per document), the following results were obtained: Approval Rate = 95.8%, MAE = 0.86, weighted MAE = 0.63, and Best Model Selection Accuracy = 95.1% (see Table 3). For individual jurisdictions, the metrics are comparable: UA (Approval 96.5%, MAE 0.87, weighted MAE 0.62, selection accuracy 95.8%) and EU (Approval 97.6%, MAE 0.89, weighted MAE 0.65, selection accuracy 97.2%). This performance profile indicates substantial alignment with the expert review process in the present evaluation setting and supports the interpretation of the pipeline as a conservative, human-supervised triage workflow.

**Table 3:** Responsible AI / legal validation metrics for UA, EU, and GLOBAL modes (*N* = 288 documents).

Evaluation mode	*N* (Docs.)	Approval rate (“Good”), %	MAE (Arbiter vs. Lawyer)	Weighted MAE	Best-model selection accuracy, %	FN_CRITICAL (missed CRITICAL), % (k/CRIT)
UA	288	96.5	0.87	0.62	95.8	11.1 (7/63)
EU	288	97.6	0.89	0.65	97.2	8.8 (6/68)
GLOBAL = max(UA, EU)	288	95.8	0.86	0.63	95.1	6.3 (5/79)

**Figure 7:**
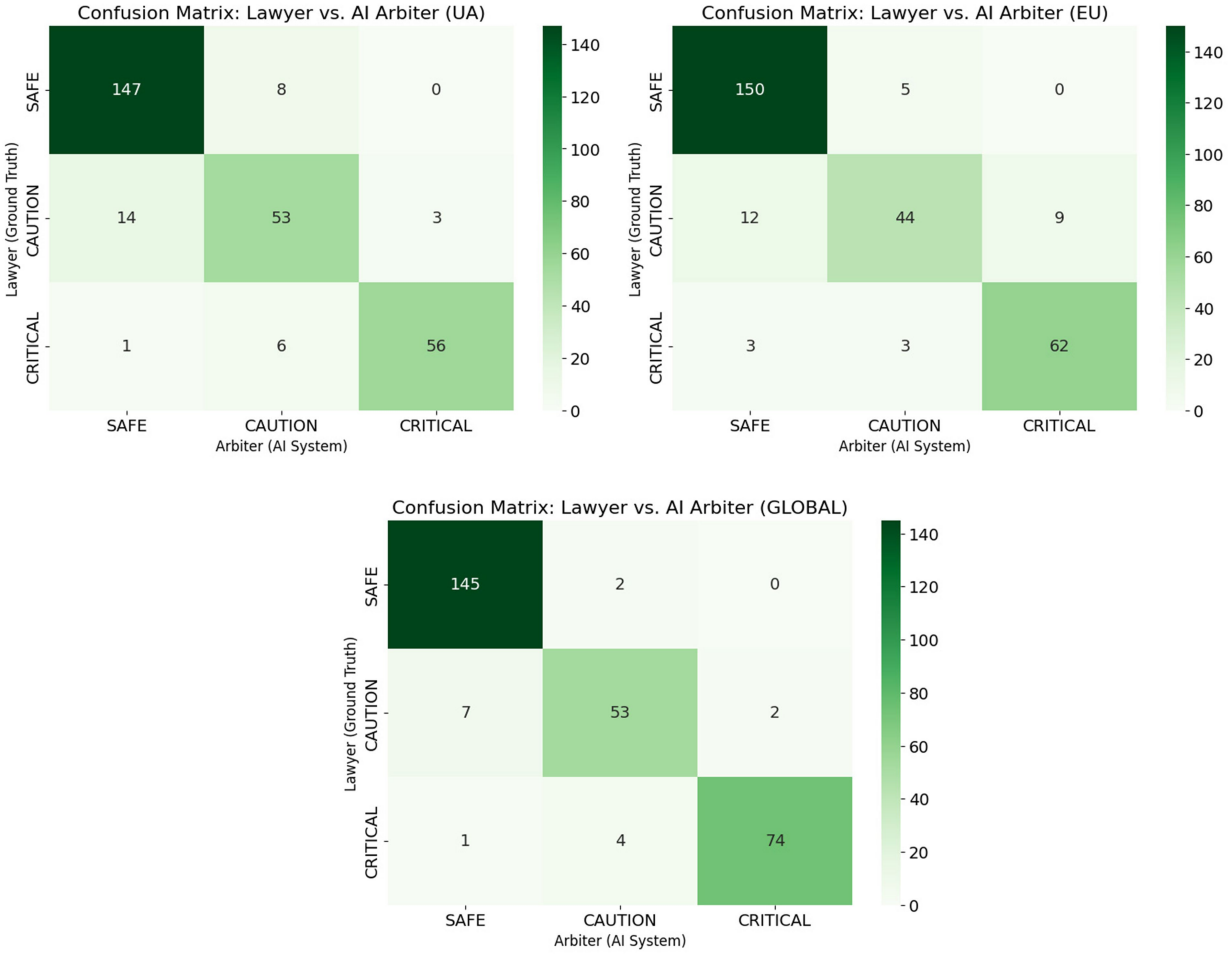
Confusion matrices between expert lawyer evaluation and arbiter decisions. **(a)** UA jurisdiction, **(b)** EU jurisdiction, **(c)** Aggregated GLOBAL mode [max (UA, EU) per document].

Approval Rate— denotes the proportion of cases in which the expert marked the evaluation as “good.” Weighted MAE— represents the MAE weighted by the lawyer’s confidence level (LAW_confidence). FN_CRITICAL is computed from the confusion matrices (Figures 7a–c) as the proportion of cases in which the lawyer classified the content as CRITICAL while the arbiter classified it as SAFE or CAUTION.

Additionally, analysis of the MAE of individual models relative to the lawyer demonstrates that the ensemble does not rely on a single dominant performer. In the GLOBAL mode, the smallest deviations were shown by DeepSeek-8B (MAE = 1.04) and Qwen-7B (MAE = 1.05), while Gemma-2B exhibited the largest deviation (MAE = 1.38). This supports the use of arbitration (median aggregation + selection of a representative rationale) as a mechanism for reducing dependence on any single model. A comparison of the MAE of individual models relative to expert assessment is presented in Table 4, substantiating the use of an ensemble approach to mitigate single-model bias.

**Table 4:** Error of individual LLMs relative to lawyer assessment (MAE vs. Lawyer) across different modes (*N* = 288 documents).

Model	MAE vs. Lawyer (UA)	MAE vs. Lawyer (EU)	MAE vs. Lawyer (GLOBAL = max)
DeepSeek-8B	0.98	1.01	1.04
Qwen-7B	1.01	1.07	1.05
Mistral-7B	1.13	1.03	1.17
Phi-3.5	1.15	1.05	1.31
Gemma-2B	1.20	1.10	1.38

Lower MAE values (see Table 4) indicate smaller deviations from expert assessment. Differences between UA/EU and GLOBAL confirm that, at the level of individual models, the error profile depends on the jurisdictional context, whereas arbitration (median aggregation + selection of a representative rationale) stabilizes the final verdict (see Table 3 and Figure 7).

For legal risk scoring, the most critical error type is the false negative (FN) for the CRITICAL tier (when the system fails to label critical material as CRITICAL). Triage quality was evaluated through “lawyer vs. arbiter” confusion matrices for UA, GLOBAL, and EU (Figures 7a–c). Visually, most of the distribution is concentrated along the diagonal (aligned classifications), while errors are predominantly localized in adjacent classes SAFE⇿CAUTION and CAUTION⇿CRITICAL, which is expected for a threshold-based scale.

The safety-oriented metric FN_CRITICAL was defined as the proportion of cases in which the lawyer classified the material as CRITICAL while the arbiter classified it as SAFE or CAUTION (Figure 7). For UA, FN_CRITICAL equals 11.1% (7/63); for EU, 8.8% (6/68); whereas in the conservative GLOBAL = max(UA, EU) mode, the indicator decreases to 6.3% (5/79). This result supports the practical value of jurisdictional aggregation as a conservative mechanism for reducing missed critical cases in the high-risk segment (Table 3).

All matrices (Figure 7) are presented using the same class scale (SAFE/CAUTION/CRITICAL), ensuring comparability across jurisdictions and the aggregated mode.

In the UA setting (Figure 7a), among CRITICAL cases identified by the lawyer, the system classifies 56 cases as CRITICAL, while 7 cases are shifted to less severe classes (1 → SAFE, 6 → CAUTION); thus, the primary risk is the “downgrading” of criticality to CAUTION.

In the EU setting (Figure 7b), CRITICAL cases align in 62 instances, while 6 cases are classified at a lower level (3 → SAFE, 3 → CAUTION).

In the GLOBAL setting (Figure 7c), the strongest safety profile is observed: CRITICAL cases align in 74 instances, with only 5 cases downgraded (1 → SAFE, 4 → CAUTION), consistent with the logic of max(UA, EU) as a “conservative” aggregation mechanism designed to minimize high-risk omissions.

From a Responsible AI perspective, the observed error structure is consistent with a conservative operating mode, especially in the GLOBAL setting, where the dominant errors occur near class boundaries rather than through systematic omission of critical cases.

Corporate Responsible AI standards require that high-impact decisions be explainable and reproducible. In the proposed pipeline, this is ensured by the fact that each verdict includes: (i) a numerical risk score, (ii) a triage class, (iii) the identifier of the model whose rationale was selected by the arbiter, and (iv) a textual rationale as an evidentiary trace. Representative audit-trail examples are provided in the previous section (Figures 3–6), where the report is presented in a format suitable for expert review (side-by-side: text → verdict → justification). Combined with fixed inference parameters and model versioning (revision pinning), this forms an accountability framework that is practically compatible with corporate requirements.

## 6 Discussion: ethics, challenges, and future trends

The proposed Ensemble & Arbiter architecture should be understood as a procedurally constrained and human-supervised workflow for legal risk triage in digital media. Rather than substituting for legal decision-making, it supports preliminary screening under conditions of epistemic uncertainty through three core safeguards: robust consensus (median aggregation), disagreement-aware review support (*σ*), and audit-oriented rationale generation. In this sense, the framework may support due diligence-oriented organizational screening across two jurisdictions, while legally consequential decisions remain with human experts and institutions.

Within this architecture, AI functions as a structured decision-support component that supports—but does not replace—legally significant decisions. The final determination regarding publication, moderation, depersonalization, or escalation remains with the human expert or organization. The human-in-the-loop protocol is intended to support procedural accountability through structured expert review and subsequent comparison with system outputs. This design helps reduce anchoring to model recommendations at the reference-evaluation stage while preserving the central role of human judgment in legally consequential decisions.

The dual-jurisdiction mode (UA vs. EU) and conservative aggregation (GLOBAL) provide a structured way to compare and combine distinct normative regimes, help reduce the risk of critical omissions, and support governance-oriented screening objectives. Audit-ready explanations and the σ-based disagreement signal function as procedural controls that make the workflow more inspectable and reviewable. From an ethical and legal perspective, this design is consistent with due diligence-oriented screening logic: AI supports structured risk assessment, while legally consequential actions remain with the organization and the expert, thereby strengthening transparency, accountability, and procedural safety.

Empirically, the human-in-the-loop validation demonstrated substantial alignment between the arbiter’s verdict and expert legal assessment in the present evaluation setting. In the GLOBAL mode = max(UA, EU), the results were MAE = 0.86, weighted MAE = 0.63, and the share of “good” evaluations exceeded 95% (Table 3). These results indicate substantial alignment with expert legal assessment, with a low average error on the 0–10 scale and a traceable audit trail: each verdict includes a numerical score, a triage class, the model selected by the arbiter, and its rationale, as illustrated in the case reports (Figures 3–6). Accordingly, “quality” in this setting is interpreted not as abstract classifier accuracy but as risk controllability in high-impact decisions, where the cost of critical omissions is disproportionately high.

The comparison of individual LLMs (Table 4) supports the view that no universally dominant model exists: the error profile varies depending on context and jurisdiction. In this setting, arbitration functions as a procedural stabilizer that helps reduce dependence on any single model. The dual-jurisdiction regime (UA vs. EU) is not merely formal: it is intended to reduce the risk of normative transfer between regulatory frameworks, while the conservative GLOBAL aggregation shows the most protective safety profile in the present evaluation. FN_CRITICAL decreases from 11.1% (UA) and 8.8% (EU) to 6.3% (GLOBAL) (Table 3), and the confusion matrices (Figures 7a–c) show that most disagreements are concentrated between adjacent classes. From a governance perspective, this error structure is preferable to systematic omission of CRITICAL cases, although boundary-level disagreements still require human review and careful policy interpretation.

A further limitation of the present study is that computational efficiency was not evaluated as a standalone deployment benchmark. The implementation was tested in a research-oriented Kaggle environment, where runtime is strongly affected by platform-specific resource constraints and is therefore not directly representative of production use. Accordingly, latency, throughput, escalation workload, and deployment-oriented scalability should be examined in future research under a more controlled serving setup.

An additional limitation is that part of the legal validation involved experts who also contributed to the design of the legal framework. While this supported doctrinal consistency and domain relevance, future work would benefit from broader external validation involving additional independent legal reviewers.

From an ethical and legal perspective, the central question concerns the boundary between automated support and human responsibility. In the proposed design, AI does not acquire legal personhood; rather, it functions as a structured decision-support component within a human-supervised screening workflow. Legally consequential action—publication, removal, depersonalization, or escalation—remains with the organization and the expert. The human-in-the-loop protocol serves as a mechanism of review, calibration, and accountability: it provides a reference framework for evaluation and helps translate model output into a structured moderation recommendation based on the principle of minimum sufficient risk mitigation. In this sense, Responsible AI is operationalized as a set of measurable process properties—expert alignment, FN_CRITICAL, auditability, and cross-jurisdiction stability—rather than as a purely declarative principle.

The limitations and future trends may be condensed into several practical theses. First, the results depend on the coverage and currency of the legal knowledge base and on the domain representativeness of the corpus (288 news items); therefore, transfer to social-media or multimodal settings requires separate validation. Second, further development should focus on risk–cost calibration of triage thresholds, formalized lifecycle governance of the legal knowledge base (including versioning and update governance), cost-sensitive safety metrics, and structured analysis of FN_CRITICAL cases. Third, future work should include stress-testing on manipulative or adversarial content, as well as broader external legal validation across a wider range of reviewers and cases. Finally, modular deployment pathways, API-oriented integration, and extension of the legal card base to additional jurisdictions should be developed so that the framework can be evaluated not only as a research prototype but also as a scalable compliance-support architecture.

## 7 Conclusion

This study presented the Ensemble and Arbiter architecture as a reproducible and human-supervised workflow for legal risk triage in digital media across two jurisdictions, Ukraine and the European Union. The proposed framework combines jurisdiction-specific legal grounding through a structured legal knowledge base, a heterogeneous ensemble of open-weight LLMs, median-based arbitration, disagreement-aware review support, and audit-oriented rationale generation. In this way, the study contributes a procedurally structured approach to preliminary legal screening in contexts where content-related decisions must be made under uncertainty, time pressure, and cross-jurisdiction regulatory complexity.

The empirical results indicate that the proposed workflow achieves substantial alignment with expert legal assessment in the present evaluation setting. In the GLOBAL mode, the system reached MAE = 0.86, weighted MAE = 0.63, an approval rate above 95%, and a lower FN_CRITICAL rate than the separate jurisdictional modes. These findings support the practical value of combining dual-jurisdiction evaluation with conservative aggregation in order to reduce missed critical cases while preserving human oversight. The results also suggest that arbitration helps reduce dependence on any single model, since no universally dominant LLM was observed across all contexts.

The practical significance of the framework lies not in replacing legal judgment, but in supporting structured, inspectable, and review-oriented triage. The system is designed to provide a preliminary risk score, a triage class, an audit-ready rationale, and a basis for escalation in cases of weak consensus or elevated risk. Accordingly, its main contribution is methodological and procedural: it offers a way to organize AI-assisted screening so that legal risk assessment remains traceable, comparable, and compatible with expert review.

At the same time, the findings should be interpreted within the limits of the present study. The evaluation was conducted on a 288-item news-oriented corpus and within a two-jurisdiction setting, which does not by itself establish broad generalizability across legal systems, media environments, or content modalities. In addition, the legal knowledge base represents the current operational scope of the framework rather than an exhaustive codification of the relevant legal regimes. Part of the legal validation also involved experts who contributed to the development of the legal framework, which supported doctrinal consistency but should be supplemented by broader external validation in future work. Computational efficiency was likewise not treated as a standalone deployment benchmark, since the implementation was tested in a resource-constrained research environment.

Future research should therefore proceed in several directions. First, the framework should be validated on broader and more diverse datasets, including social-media, multimodal, and adversarially manipulated content. Second, the legal card base should be extended and maintained as an updatable module, including versioning and formalized update governance. Third, deployment-oriented studies should examine latency, throughput, escalation workload, and API- or plug-in-based integration in more controlled serving environments. Fourth, additional comparative experiments should assess alternative arbitration rules, threshold calibration strategies, and safety-oriented error trade-offs. Through these extensions, the proposed framework can be developed further as a scalable compliance-support architecture for high-risk digital-media screening under human responsibility and institutional oversight.

Reproducible computational notebook (Kaggle) for the experimental workflow is available online at: https://www.kaggle.com/code/khrystynalip/notebookbab4261945.

## Data Availability

The datasets presented in this study can be found in online repositories. The names of the repository/repositories and accession number(s) can be found below: The datasets supporting the findings of this study are publicly available. The “News dataset about Ukraine” is available on Figshare: https://doi.org/10.6084/m9.figshare.29020670.v3. The “EU–Ukraine Legal Keyword Knowledge Base for Content Risk Triage (CSV)” is available on Figshare: https://doi.org/10.6084/m9.figshare.31386106.v1. A reproducible analysis notebook is available on Kaggle: https://www.kaggle.com/code/khrystynalip/notebookbab4261945.
